# Prevalence and risk factors for extended-spectrum β-lactamase producing antimicrobial-resistant *E. coli* in urinary tract infections among inpatients in the tertiary hospitals in Zanzibar (Tanzania): a prospective cross-sectional study

**DOI:** 10.11604/pamj.2024.47.193.37920

**Published:** 2024-04-16

**Authors:** Muhiddin Hamada Omar, Andrew Martin Kilale, Huba Khamis Rashid, Elibariki Reuben Mwakapeje, Isaac Manase Onoka, Angaza Amos Gimbi

**Affiliations:** 1 Department of Preventive Service and Health Education, Ministry of Health, Unguja, Zanzibar, Tanzania; 2 The Open University of Tanzania, Dar es Salaam, Tanzania; 3 National Institute for Medical Research, Muhimbili Research Centre, Dar es Salaam, Tanzania; 4 Department of Pharmacology, School of Health and Medical Science, State University of Zanzibar, Zanzibar, Tanzania; 5 Food and Agriculture Organization of the United Nations, emergence Center for Transboundary Animal Diseases, Dar es Salaam, Tanzania; 6 Department of Chemistry, University of Dodoma, Dodoma, Tanzania

**Keywords:** *E. coli*, extended-spectrum β-lactamase, antimicrobial, resistance, urinary tract infection, prevalence, risk factors

## Abstract

**Introduction:**

Extended-spectrum β-lactamase (ESBL) production among Enterobacteriaceae, such as E. coli, has been increasing worldwide, which causes treatment failure for urinary tract infections. Therefore, this study aimed to determine the prevalence and risk factors for the production of ESBL in E. coli from patients with urinary tract infections (UTI) in Zanzibar.

**Methods:**

a prospective cross-sectional study was conducted from January 2018 to December 2021 in Zanzibar. Data were retrieved from a routine bacteriological laboratory culture report from urine samples of 4306 patients at the Lancet Laboratory. In addition, the patient's social demographics and clinical data were retrieved by examining the medical records in the respective hospitals. All inpatients older than fifteen years diagnosed with urinary tract infections (UTI) and requested urine culture and sensitivity were included. The Chi-square and Fischer's exact tests were used to compare antibiotic resistance. In addition, a binary logistic regression analysis was used to predict ESBL production risk factors.

**Results:**

the prevalence of E. coli-producing ESBL was 13.4% (578/4030). Infection of ESBL. E. coli was prevalent in females 52.6% (n=304) compared to male patients, 47.4% (n=274), and the majority 38.8% (n=224), were people of young age, between 16-30 years. The average age of patients was 31.5±10.2 years, with minimum age of 16 years and a maximum age of 72 years. In multivariate analysis, results shown that previously hospitalised patients aOR: 6.35, 95% Cl 3.37-11.92; p=0.001, long hospital stays aOR: 10.34, 95% Cl 3.03-22.29; p <0.001, prior use of penicillin aOR: 7.78, 95% Cl 2.99-29.11; p < 0.001, and prior use of cephalosporin drugs aOR: 4.64, 95% Cl 2.99-9.96; p=0.001, were strongly associated with the emergence of ESBL-producing E. coli in urinary tract infection patients. ESBL E. coli showed high resistance to amoxicillin 99.5% (n=575), ampicillin 97.8.% (n=570), cotrimazaxole 86.2% (n=344), ceftriaxone 73.7% (n=344), ciprofloxacin 73.2% (n=423), and ceftaxime 59.5% (n=426). There was a less resistance to ampicillin -cloxacillin 44.3% (n=256), gentamicin 22.5% (n=22.5), and norfloxacin 18.9% (n=109) respectively. Isolates were shown to be more susceptible to meropenem at 1.6% (n=9).

**Conclusion:**

the overall prevalence of ESBL-producing E. coli is 13.4%. The risk of emergence ESBL was higher in patients with previous history of hospitalisation, long hospital stay, prior use of penicillin and cephalosporin drugs. High level of antimicrobial resistance observed against most commonly used antibiotics in treatment of urinary tract infections. The clinicians should rely on microbiological diagnosis in treatment of UTIs to reduce risk of treatment failure. Further study should be carried out to assess the prevalence and resistance pattern of other uropathogens and other risk factors.

## Introduction

Urinary Tract infections (UTIs) due to *Escherichia coli* (*E. coli*) are the most common infectious diseases [1,2]. Prolonged urinary tract infections can result in patient morbidity if not adequately treated [3]. The emergence of the ESBL *E. coli* strain increases the incidence and complication of managing UTI patients [4]. Furthermore, it leads to long-term hospitalisation and increases the cost of treatment [4,5]. Extended Spectrum β-lactamase-producing bacteria harbours ESBL enzymes that hydrolyse β-lactam antibiotics and associated with the treatment failure of β-lactam antibiotics [5,6].

Early laboratory diagnosis and clinical management of UTIs, can limit the incidence of complications in developing countries [7]. However, in Zanzibar, lacking a standard laboratory for bacterial culture and sensitivity and results many UTI cases undiagnosed with specific concerns to ESBL producers, including *E. coli*. Therefore, the prescription for treating infectious diseases, including UTI, is done empirically. Inappropriate antibiotic prescriptions may encourage the emergence of resistant ESBL-producing *E. coli* [8,9]. This leads to increasing antimicrobial resistance and difficulty in treating infections associated with ESBL-producing *E. coli* [10].

The occurrence of ESBL-producing *E. coli* in the urine of inpatients with UTI has been reported in several studies worldwide [5,11]. Zanzibar experienced an increasing prevalence of UTIs. However, the magnitude of the UTI associated with ESBL-producing uropathogens with particular concern to *E. coli* is unclear. Therefore, this study has been conducted to investigate the prevalence of ESBL-producing *E. coli* in patients hospitalised for treatment with urinary tract-associated infections. Additionally, examine the magnitude of antimicrobial-resistant ESBL-producing *E. coli* from UTI patients. The findings of this study will be useful in the management of ESBL-producing *E. coli* associated with UTI and also improvement of treatment outcomes for patients with UTI in Zanzibar.

## Methods

The prospective cross-sectional study design was employed covering four consecutive years, from January 2018 to December 2021. The study involved inpatients hospitalized at the tertiary private and public hospitals diagnosed with UTIs and requested bacteriological culture and sensitivity. Data were collected during routine bacteriological culture at the Lancet Microbiological Laboratory. This laboratory serves as an outsourced laboratory for private and public hospitals in Zanzibar and is responsible for performing bacteriological culture and sensitivity. In addition, the patient's social demographic and clinical data were retrieved by examining the medical records in the respective hospitals.

**Study population:** all inpatients older than fifteen years, who were diagnosed with UTI and requested urine samples for culture and sensitivity at Lancet Microbiological Laboratory. The average age of patients is 31.5± 10.2 years. Over 4 years, urine samples from 4306 patients, including 1993 male and 2313 female were received in the laboratory for bacteriological culture and drug sensitivity.

**Data collection:** all laboratory procedures were done following standard operating procedures and Clinical Laboratory Standards Institute (CLSI) guidelines [12]. The urine samples were received from four purposively selected tertiary hospitals in Zanzibar (Mnazi Mmoja, Al- Rahma, Nampola and Tawakal Hospital). The patient's information was retrieved from the sample request form upon arrival in the laboratory. Urine collection, storage, and transportation were done in adherence to the laboratory's standard operating procedures [13]. A total of 4306 non-duplicate midstream urine samples were collected. Urine samples were cultured on a routine basis by semi-quantitative methods [13]. Conventional microbiological methods identified the ESBL-producing *E. coli* isolate. Moreover, screening of ESBL-producing *E. coli* was carried out through a zone of inhibition of ≤25mm for ceftriaxone and/or ≤22mm for ceftazidime and or ≤17 mm cefpodoxime and or 27mm for cefotaxime considered as positive ESBL producer [12] ESBL-producing *E. coli* was confirmed phenotypically using the combined disc method. Discs containing ceftazidime (30µg) and a combination of discs containing clavulanic acid (30µg + 10µg) were placed at 25mm apart. The isolate was considered an ESBL producer when an increase in zone of the diameter of ≥5mm in the zone of inhibitor for ceftazidime+ clavulanic acid compared to ceftazidime alone was confirmed as ESBL-producing *E. coli* according to CLSI guideline [13]. For quality assurance, the reference strains of *E. coli* (ATCC 26122) and Klebsiella (ATCC 75674) were used in quality control for culture and susceptibility tests as well as for the detection of ESBL detection.

Antibiotics susceptibility test was carried out using Kirby-Bauer's disc diffusion method, and interpretation of the results, whether resistant, susceptible or intermediate, were based on recommendations by Clinical and Laboratory Standard Institute guidelines [12].

**Data analysis:** the data were analysed using Statistical Package for Social Science version 21 (Chicago Inc). A descriptive analysis was performed, and information was summarised using frequency and percentage. The differences in antibiotic resistance to ESBL and non-ESBL *E. coli* were compared using the Chi-square test and Fischer's exact test. Multivariate analysis by binary logistic regression was performed. The Adjusted Odds ratio (aOR) and 95% confidence interval (95%Cl), were used to show the association between antibiotics resistance with ESBL and non-ESBL *E. coli*

**Ethical considerations:** the Lancet laboratory and respective hospitals granted permission to collect and use the data. The study was carried out after ethical approval, which was obtained from the Zanzibar Health Research Ethical Review Committee of the Zanzibar Health Research Institute with approval number ZAHREC/O4/ST//33.

## Results

The annual frequency distribution of ESBL-producing *E. coli* is shown in Table 1. Over the 4 years, there was a constant increase in ESBL *E. coli* isolated urine samples. Annual frequency was 17.6% in 2018, 21.5% in 2019, and 26.8% in 2020 and 34.0% in 2021 respectively (P-value > 0.05). The prevalence of ESBL-producing *E. coli* is shown in Table 2. Overall, urine samples from 4306 patients, were received and cultured. A total of 74.9% (n=2248) were *E. coli*, out of which 25.7% (n= 578) were confirmed as ESBL-producing *E. coli*. The prevalence of ESBL-producing *E. coli* was 13.4% (578/4030). Based on demographical and social characteristics of the patients' prevalence of ESBL and non-ESBL-producing *E. coli* from the urinary tract infections shown in Table 2.

**Table 1:** distribution of ESBL E. coli reported in clinical laboratory according to year

Year	ESBL *E. coli* frequency N= 578	Yearly comparison	P-value
2018	102(17.6)	2018-2019	0.0028
2019	124(21.5)	2019-2020	0.001
2020	155(26.8)	2020-2021	0.005
2021	197(34.0)	2021-2018	0.006

**Table 2:** prevalence of ESBL non-ESBL-producing E. coli from urinary tract infection based on demographic and social factors

Characteristics		ESBLs *E. coli* N=578(25.7%)	Non ESBLs *E. coli* N=1670(74.3%)	P value
Age	< 15	123(21.3)	402(24.1)	<0.05
	16-30	224(38.8)	658(39.6)	0.05
	31-45	175(12.9)	265(15.9)	>0.05
	>45	162(28.3)	342(20.5)	>0.05
Gender	Male	274(47.4)	705(42.2)	0.49
	Female	304(52.6)	965(57.8)	

Most of the patients infected by ESBL *E. coli* were females, 52.6% (n= 304), compared to males 47.4% (n=274) and the majority 38.8% were people of young age, between 16-30 years. The average age of the study participants was 31.5± 10.2 years, with a minimum age of 16 years and maximum age of 72 years. The isolates were highly reported between ages 16-30 (p-value = 0.05). Based on clinical characteristics, the prevalence of ESBL-producing *E. coli* is reported in Table 3. In univariate and multivariate analysis in (Table 3), a statistically significant association was found in patients with previous hospitalisation aOR: 1.56, 95% Cl 12-2.24; p <0.001, long hospital stay aOR: 10.34, 95% Cl 3.03-22.29; p <0.001, prior use of penicillin aOR: 3.10, 95% Cl 3.2-27.7; p <0.001, prior use of cephalosporin drugs aOR: 2.68, 95% Cl 1.69-4.25; p=0.001). However, no statistically significant difference was found in the relationship between prior use of fluoroquinolone drugs aOR: 1.68, 95% Cl 0.68-4.01; p=0.23 and foley catheterisation and the emergence of ESBL *E. coli* aOR: 0.80, 95% Cl 0.78-3.94; p=0.17. In multivariate analysis (Table 3), statistically, results showed a strong significant association for the emergence of ESBL-producing *E. coli* among urinary tract infection patients in case of previously hospitalised aOR: 6.35, 95% Cl 3.37-11.92; p=0.001, long hospital staying (aOR: 2.93, 95% Cl 1.88-4.89; p < 0.001, prior use of penicillin drugs aOR: 7.78, 95% Cl 2.99-29.11; p < 0.001, and prior use of cephalosporin drugs aOR: 4.64, 95% Cl 2.99-9.96; p=0.001.

**Table 3:** prevalence of ESBL and non-ESBL-producing E. coli from urinary tract infections based on clinical characteristics (univariate and multivariate analysis)

Characteristics		ESBLs *E. coli* N=578 (25.7%)	Non ESBLs *E. coli* N=1670 (74.3%)	Univariate analysis		Multivariate analysis	
				aOR (95%Cl)	P-value	aOR (95%Cl)	P=value
Previous hospitalisation	No	124(21.4)	769(46.0)	**Ref**		6.35(3.37-11.92)	0.001
	Yes	454(78.6)	901(54.0)	3.8(2.22-9.01	<0.001		
Recurrent UTI	No	135(23.4)	695(41.6)	Ref			
	Yes	443(76.6)	975(58.4)	1.68(0.60-2.04)	0.56	-	-
Long hospital stay	No	200(34.6)	1259(74.9)	**Ref**			
	Yes	378(65.4)	420(25.1)	10.34(3.03-22.29)	<0.001	2.93(1.88-4.89)	<0.001
Foley catheterization	No	552(93.9)	1460(87.4)	**Ref**			
	Yes	26(6.1)	210(12.5)	0.80(0.78-3.92)	0.17	-	-
Prior use of penicillin	No	199(36.6)	1050(62.9)	**Ref**			
	Yes	379(64.4)	620(37.1)	3.10(3.2-27)	<0.001	7.78(2.99-29.11)	<0.001
Prior use of Fluoroquinolone	No	365(63.1)	930(56.0)	**Ref**			
	Yes	231(36.9)	740(44.3)	1.68(0.68-4.01)	0.23	-	-
Prior to the taking of cephalosporin	No	417(72.1)	894(53.5)	**Ref**			
	Yes	161(27.9)	776(46.5)	2.68.0(1.69-4.25)	<0.001	4.67(2.99-9.96)	0.001

A comparison of the antibiotic resistance pattern revealed an association between antimicrobial resistance with ESBL production in *E. coli* pathogens (Table 4). Antimicrobial resistance was reported to common classes of antibiotics except for meropenem. All isolates showed a high antimicrobial resistance to commonly used antibiotics. The ESBL *E.coli* showed high level of resistance to amoxicillin with resistance rate, 99.5% (n =575) (P=0.001, followed by ampicillin 97.8% (n=570) (P= 0.02), cotrimazaxole 86.2% (n=498) (P= 0.03), ceftriaxone 73.7% (n= 344) (P=0.05), ciproflaxin 73.2% (n=423) (P= 0.01) vis Non ESBL *E. coli*, 85.2% (n=1423) (P= 0.003), 79.4% (n=1330) (P= 0.003), 60.2% (n= 1005) (P= 0.02), 75.6% (n=1263) (P= 0.04), 57.3% (n=96) (P= 0.003 respectively.

**Table 4:** antibiotics resistance pattern between ESBL and non-ESBL-producing *E. coli* from urinary tract infection

Antimicrobial agents	ESBLs *E. coli* (N= 578)				Non ESBLs *E. coli* (N= 1670)			
	Susceptible (%)	Resistance (%)	χ^2^	P-value	Susceptible (%)	Resistance (%)	χ^2^	P-value
Ampicillin	8(2.2)	570(97.8)	7.08	0.002	340(20.6)	1330(79.4)	2.99	0.003
Amoxacillin	3(0.5)	575(99.5)	7.42	0.001	247(14.8)	1423(85.2)	5.24	0.002
Norfloxacin	469(81.1)	109(18.9)	6.86	0.170	1325(79.4)	345(20.6)	5.24	0.150
Ciproflaxin	155(26.8)	423(73.2)	0.85	0.010	708(42.7)	962(57.3)	1	0.003
Nalidixic Acid	282(48.8)	296(51.2)	8.37	0.200	1208(72.4)	462(27.6)	7.81	0.060
Meropenem	569(98.4)	9(1.6)	7.11	0.002	1670(100)	0.0(00)	-	-
Cotrimaxazole	80(13.8)	498(86.2)	15.4	0.030	665(39.8)	1005(60.2)	12.58	0.020
Cefriaxone	152(26.3)	344(73.7)	19.66	0.050	407(24.4)	1263 (75.6)	11.33	0.010
Ceftaxime	234(40.5)	426(59.5)	5.68	0.040	774(46.4)	896(53.6)	2.08	0.020
Amp-clox.	322(55.7)	256(44.3)	14.4	0.010	1293(77.1)	377(22.9)	11.75	0.002
Gentamicin	448(77.5)	130(22.5)	9.65	0.100	1263(75.5)	407(24.5)	4.65	0.070

AMP = Ampicillin; AMC = amoxacillin CRO = Ceftriaxone; CTM = Cotrimaxazole, CIP = Ciprofloxacin; GN = Gentamicin; CTX = Cefotaxime; NOR = Norfloxacin; NAL = Nalidixic Acid; CHL = Chloramphenicol; MEM = Meropenem; and AMPC = Ampicloxacillin

Less resistance was observed to ESBL *E. coli* for ceftaxime 55.7% (n=322) (P= 0.04), nalidixic acid 51.2% (n=296) (P= 0.20), ampicillin- cloxacillin 44.3% (n=256) (P=0.01) vis Non ESBL *E. coli*, (53.6% (n=896) (P=0.020), 27.6% (n=492) (P=0.06), 22.9% (n=377) (P= 0.01) respectively. All isolates were shown susceptibility to meropenem 1.6% (n=9) (P= 0.002), gentamicin 22.5% (n=130) (P=0.10, 20.6% (n=345) (P=0.06) for ESBL *E. coli* vis (0.0%), 24.5% (n=407) (P=0.07) and norfloxacin 18.9% (n=409) (P=0.2) for Non ESBL *E. coli* respectively. All isolates 100% (n=578) of ESBL *E. coli* showed a high multi-drug resistance (MDR) level. Among 1670 Non-ESBL, *E. coli* showed MDR phenotypes in 10 of 11 antibiotics tests.

All isolates 100% (n=578) of ESBL *E. coli* showed a high multi-drug resistance (MDR) level. Among 1670 Non-ESBL, *E. coli* showed MDR phenotypes in 10 of 11 antibiotics tests.

## Discussion

The study aimed to investigate the prevalence and risk factors for ESBL-producing *E. coli* in patients hospitalised for urinary tract associated infections and their resistance to antibiotics. The isolates were highly resistant to most commonly used antibiotics. We found that the prevalence of ESBL-producing *E. coli* was 13.8%. The results suggested that the age 15-30 years were more risk group for infection of UTI. We also observed that the previous hospitalization, long hospital stays, prior use of penicillin and cephalosporin drugs were statistically significant for the emergence of ESBL *E. coli* among UTIs patients. High level of resistance observed to most common used antibiotics.

In the present study, the reported prevalence of ESBL-producing *E. coli* was high (13.8%). This is similar to study conducted in Korea with prevalence 12.1% [11]. However, the finding is lower than the study in Iran, 67.7% [14]. This variation might be attributed to geographical location and the hygienic status of the community in particular countries. A high proportion of ESBL-producing *E. coli* was isolated in the age group between 15- 30 years compared to others. It is in line with studies from Nepal [7] and Ethiopia [6,15]. However, this contradicts with study done in Palestine [16]. This is the most reproductive age hence the risk of infection with UTI is high. In addition, a high proportion of ESBL-producing *E. coli* was reported in female compared to male patients. This finding is similar to the study in Saudi Arabia [17]. However, statistical significance in male than female patients was found in other study [18]. This gender-based variation in the proportion of ESBL-producing *E. coli* to UTI patients is associated with the genital anatomical nature of the urinary tract of females [19]. Women have short urethra hence is easy for uropathogens to reach the internal surface of the female reproductive tract [20,21].

In this study, ESBL-producing *E. coli* was higher in previously hospitalised patients than in patients who had never been hospitalised before. The results agree with the study conducted in Mexico [22] and in Tanzania [23]. The previous hospitalisation increases the risk of the spread of resistant infectious pathogens [20]. Therefore, improving infection prevention and control measures in hospital settings is essential.

Previous studies conducted elsewhere showed that, the emergence of the ESBL producing *E. coli* in patients with previous hospitalisation, recurrent UTI, Long hospital stay, use catheter, prior use of antibiotics, inpatient UTI, past history of urogenital operation and female gender [11,20,23]. In the present study, univariate analysis showed that previous hospitalization, recurrent UTI, Long hospital stay, use of catheter, prior use of penicillin, cephalosporin and floroquinolone contributed to the emergence of the ESBL producing *E. coli* (Table 3). However, the multivariate logistic regression analysis showed that prior use of penicillin was a strong significant risk factor for the emergence of the ESBL among *E. coli*, followed by previous hospitalization and prior use of cephalosporin antibiotics. The recurrent UTI (p=0.17), prior use of floroquinolone (p=0.23) and use catheter (p=0.56) were not risk factors for the emergence of the ESBL *E. coli* in urinary tract infection patients (Table 3). Several studies showed that use of catheter, recurrent UTI and prior use of floroquinolone, were strongly associated with emergence of ESBL *E. coli* [11,19]. However, the study found no association with the emergence of ESBL producing *E. coli*. Our study showed that the risk for emergence of ESBL producing *E. coli* was 7 times higher in those patients with prior use of penicillin and 6 times higher to those patients with previous history of hospitalization. Therefore, it is considered the most strongest risk factor for the emergence of the ESBL-producing *E. coli*. This is supported with other studies [21,24,25]. This is attributed with the unrestricted availability of penicillin antibiotics over the counter including amoxicillin and ampicillin in community pharmacies. Here community awareness on antibiotics resistance and antibiotics use is needed as well as over the counter availability of the antibiotics should be restricted. The association of previous hospitalization and the emergence of the ESBL producing *E. coli* in our study is in agreement with a previous study by Taha *et al*. 2018 [19]. This implies the need to reduce risk of transmission of hospital acquired infections through improvement of the infection and prevention control mechanisms in hospital settings.

This study has found that using third generation beta lactam antibiotics including cephalosporin, drugs prior encounter with a urinary tract infection could increase the development of ESBLs among *E. coli* as the strong risk factor [26], which is in agreement with our study. The empirical treatment of UTI to cephalosporin antibiotics to admitted patients without laboratory diagnosis, is the most common practices among the prescribers in many health facilities [27]. Definitely irrational prescription, increases the risk for the emergence of ESBL producing *E. coli* hence needs for establishment of the hospital antimicrobial stewardship committee to monitor antibimicrobial use is important for rational use of antibiotics for prophylaxis and treatment. Foley catheterisation and recurrent UTI reported in other studies, as among the factors linked to the emergence of ESBL-producing *E. coli* in urinary tract infections [19,28]. Our study found no significant association between Foley catheterisation and recurrent UTI and the development of ESBL *E. coli* Poor unhygienic practices of Foley catheter insertion probably contribute to the cross-contamination of ESBL *E. coli* producers [26]. The infection is likely to occur through direct contact with poor sterilised hands with a collecting system through the lumen of the catheter [29]. Therefore, need to improve sterilisation measures during catheterisation. This finding is a wake-up call to physicians to consider the risk factors when diagnosing a patient with UTI before prescribing antibiotics to enhance the empirical UTI treatment. Moreover, recurrent UTI, leads to frequent use of antibiotics to patients. This indicates needs to improvement of community hygiene and completion of entire course of dose of antibiotics.

These studies found that isolates were highly resistant to commonly used antimicrobial agents to treat urinary tract infections [30]. All isolates tested positive for ampicillin, amoxicillin, co-trimaxazole, and ciprofloxacin resistance. This finding is consistent with findings from studies [15,29] which were reported 90% (n= 89) amoxicillin, co-trimaxazole 62.11% (n=59) and ciprofloxacin 61.05% (n= 58). In addition, the study reported the association between increasing incidence of infections and over-prescription of β-lactam antibiotics [17,28,29]. This induces selective pressure among pathogens hence scaling up the resistance [22,30]. In addition, these antibiotics are drugs of choice for UTIs and are frequently prescribed in hospitals and are easily dispensed and available over the counter in most community pharmacies [22,27]. Susceptibility to ampicloxacillin is consistent with the study [30]. This is because used as an inhibitor against the activity of ESBL enzyme produced by most ESBLs producers, including *E. coli* nalidixic acid and gentamycin were also more susceptible to ESBL-producing *E. coli*. This might be because it is available in the injectable formulation; therefore, it is not likely available over-the-counter [26]. In our study, none of the isolates showed resistance to meropenem, and the drug was the most effective against ESBL producers. This is because it cannot be administered as an empirical drug unless the infection is life-threatening. Moreover, it is stable against the hydrolysis effect of ESBL enzymes produced by ESBL producer's bacteria and is not frequently prescribed in many hospitals. Therefore, call physicians to rely on laboratory results to avoid the wrong prescription of antibiotics. This is the first study to provided valuable information for monitoring of the spreading of the ESBL-producing *E. coli* associated with urinary tract infection as well as antimicrobial resistance at the hospital settings in a study area. The limitation of our study is to use prospective data which might have prevented the establishment of the association of the emergence of ESBL *E. coli* in urinary tract infection with additional clinical risk factors.

## Conclusion

In conclusion, the chance of the emergence of the ESBL producing *E. coli* in urinary tract infections appears to have increased in four years consecutively. This influences the rate of use of antibiotics for the management of UTI. The overall prevalence of ESBL producing *E. coli* in urinary tract infections was 13.8%. The risk for the emergence of the ESBL producing *E. coli* in urinary tract infections seems to be increases in case of prior use of penicillin, previous hospitalization and prior use of cephalosporin and long hospital stay. Hence the clinicians should consider these risk factors in management of the UTIs. The isolates had high resistance rate to penicillin, cephalosporin, fluoroquinolone and sulfamethoxazole. Hence, these drugs should not be used as empirical treatment against UTI associated with ESBL producing *E. coli*. All isolates were more susceptible to carbapenem (meropenem) and aminoglycoside (gentamicin), hence these drugs were most effective antibiotics against ESBL producing *E. coli* Establishing hospital hospitals and community antimicrobial stewardship is critically essential to reduce irrational use of antimicrobials drugs to improve treatment outcomes Furthermore, improving infection and prevention control measures and hygiene is necessary to reduce the spread of pathogens in the hospital settings. The hospital should make efforts by provide medical education to clinicians to ensure adherence to the guidelines for rational antibiotics prescription and urinary catheterization.

### What is known about this topic

- *The occurrence of ESBL-producing E. coli in the urine of inpatients with UTI is a global public health threat;*
- *Antimicrobial resistance due to ESBL-producing E. coli threatens the treatment of urinary tract infections;*
- *Poor diagnostic facilities and drug misuse have scaled up the antimicrobial resistance*.

### What this study adds

- *The prevalence of UTI due to ESBL-producing E. coli has been increasing in Zanzibar;*
- *The reported antimicrobial resistance due to ESBL-producing E. coli is very high;*
- *The study found that previous hospitalisation, extended hospital stay, and prior use of penicillin and cephalosporin drugs are strong risk factors for the emergence of ESBL-producing E. coli in UTI patients*.
